# Assembly and regulation of the chlorhexidine-specific efflux pump AceI

**DOI:** 10.1073/pnas.2003271117

**Published:** 2020-07-07

**Authors:** Jani Reddy Bolla, Anna C. Howes, Francesco Fiorentino, Carol V. Robinson

**Affiliations:** ^a^Physical and Theoretical Chemistry Laboratory, Department of Chemistry, University of Oxford, Oxford OX1 3QZ, United Kingdom

**Keywords:** mass spectrometry, chlorhexidine, efflux pumps, transcriptional regulator

## Abstract

Few antibiotics are effective against *Acinetobacter baumannii*, one of the most successful pathogens responsible for hospital-acquired infections. Resistance to chlorhexidine, an antiseptic widely used to combat *A. baumannii*, is effected through the proteobacterial antimicrobial compound efflux (PACE) family. The prototype membrane protein of this family, AceI (*Acinetobacter* chlorhexidine efflux protein I), is encoded for by the *aceI* gene and is under the transcriptional control of AceR (*Acinetobacter* chlorhexidine efflux protein regulator), a LysR-type transcriptional regulator (LTTR) protein. Here we use native mass spectrometry to probe the response of AceI and AceR to chlorhexidine assault. Specifically, we show that AceI forms dimers at high pH, and that binding to chlorhexidine facilitates the functional form of the protein. Changes in the oligomerization of AceR to enable interaction between RNA polymerase and promoter DNA were also observed following chlorhexidine assault. Taken together, these results provide insight into the assembly of PACE family transporters and their regulation via LTTR proteins on drug recognition and suggest potential routes for intervention.

## Significance

*Acinetobacter baumannii* has become challenging to treat due to its multidrug resistance mediated by active drug efflux pumps. The prototype member of the proteobacterial antimicrobial compound efflux (PACE) family, AceI of *A. baumannii*, is implicated in the transport of widely used antiseptic chlorhexidine, while AceR is associated with regulating the expression of the *aceI* gene. Here we apply native mass spectrometry to show that AceI forms dimers at high pH, and that chlorhexidine binding facilitates the functional form of the protein. Also, we demonstrate how AceR affects the interaction between RNA polymerase and promoter DNA both in the presence and in the absence of chlorhexidine. Overall, these results provide insight into the assembly and regulation of the PACE family.

Chlorhexidine is a broad-spectrum antiseptic agent that has been used extensively in human healthcare and veterinary medicine for many decades, mainly for hand hygiene, in alcohol-based antiseptics, in mouthwashes, and in hospitals during surgery (1). Its antibacterial mechanism of action is described as a disruption of bacterial membranes, with subsequent leakage of cytoplasmic components (2, 3). Chlorhexidine is most active toward a wide range of gram-positive bacteria but also has activity against gram-negative bacteria and fungi and can be used with a variety of common antibiotics (4). However, its widespread application for almost 60 y, in clinical and domestic settings, has imposed selective pressure and accelerated the emergence of resistant strains among pathogenic bacteria, including *Acinetobacter baumannii*, *Pseudomonas aeruginosa*, and *Klebsiella pneumoniae* (1).

The primary resistance mechanism of chlorhexidine can be attributed to the expression of several types of multidrug efflux pumps in various bacteria (5). These include members of the following families: MATE (multidrug and toxic extrusion), ABC (ATP-binding cassette), MFS (major facilitator superfamily), RND (resistance-nodulation-division), SMR (small multidrug resistance), and PACE (proteobacterial antimicrobial compound efflux) (5).

The first transporter protein of the PACE family, AceI (*Acinetobacter* chlorhexidine efflux protein I), was identified during a transcriptomic study in *A. baumannii*. Subsequently, it has been shown to confer resistance to chlorhexidine, mediated by an active efflux mechanism (6). *A. baumannii*, a gram-negative opportunistic human pathogen, is one of the six most important multidrug-resistant microorganisms in hospitals worldwide (7). The PACE family consists of proteins of ∼150 amino acid residues with four transmembrane helices (*SI Appendix*, Fig. S1*A*). Studies of AceI homologs from other species have expanded the substrates for PACE family proteins to include synthetic biocides. These molecules include benzalkonium, acriflavine, proflavine, and dequalinium in addition to chlorhexidine (8) and, more recently, naturally occurring short-chain diamines (9).

Transport experiments conducted in whole cells and proteoliposomes suggest that the prototype protein AceI use electrochemical proton gradient to translocate substrates (6, 9). AceI shares a similar size and predicted secondary structure as the SMR family, to date but no structural or functional similarities have been described for these two families. However, AceI does share a conserved glutamic acid residue located in the middle of the first transmembrane helix. Mutation of this glutamate residue to glutamine renders the protein unable to mediate chlorhexidine transport or to confer resistance, although the protein does retain binding affinity for chlorhexidine (6). This suggests that this residue is involved in an aspect of transport unrelated to substrate binding, possibly an ion-coupling reaction. However, the mechanistic details of how this anionic residue couples efflux with the pH gradient have not yet been elucidated.

The expression level of AceI is known to be controlled at the transcriptional level by a regulatory protein, AceR (10). AceR belongs to the large family of LysR-type transcriptional regulators (LTTRs) in bacteria. The proteins in this family are ∼300-aa residues long with a poorly conserved C-terminal inducer-binding domain and a well-conserved DNA-binding domain at the N terminus (11). Typically, LTTR proteins require binding of a small molecule (an inducer) to activate transcription. LTTR proteins generally form dimers, tetramers, or octamers in solution but typically bind to DNA as tetramers (11–13). In these complexes, the LTTR binds the divergent promoter region on one or more sites commonly designated recognition binding sites (RBS) and activation binding sites (ABS) (11, 13–16). In the absence of small molecule inducers, LTTRs bind the RBS and ABS sites simultaneously, causing the DNA to bend. However, this DNA binding is not sufficient for transcriptional activation and requires an inducer molecule to activate transcription. Inducer binding to the LTTR has been proposed to evoke a quaternary structural change resulting in a shift of the DNA-binding site, probably via a sliding dimer mechanism (17). This results in a relaxation of DNA bending and allows RNA-polymerase (RNAP) binding to the promoter to initiate transcription (18–20). How the ligand-induced conformational changes activate transcription remains elusive, however, due mainly to the lack of structural information about DNA-bound full-length LTTRs and key interactions of LTTRs with RNAP. Moreover, the exact mechanism of transcriptional activation by LTTRs remains a subject of active investigation.

The AceR transcriptional regulatory protein is encoded divergently to the *aceI* gene. Knockout studies have shown that chlorhexidine-induced expression of *aceI* was abolished in a Δ*aceR* strain, and a DNase I footprinting assay identified two AceR-binding sites within the *aceR*/*aceI* promoter region (10). The same study showed that the inducer-binding domain of AceR binds to chlorhexidine; however, little is known about AceR function in terms of the oligomeric state, promoter recognition, interaction with chlorhexidine, and communication with RNAP.

The PACE family of transporters has been reported only recently, and high-resolution atomic structures and mechanistic details remain elusive. Moreover, the mechanism of PACE family regulation by regulatory proteins is not fully understood. Here we used native mass spectrometry (MS) optimized for the analysis of soluble and membrane protein complexes and their interactions with nucleic acids, lipids, and drugs over a range of different solution conditions (12, 21–32). Using this approach, we probed changes mediated by chlorhexidine binding to AceI, and a key mutant, as well as to AceR, and examined their impact on DNA and RNAP interactions.

## Results and Discussion

### AceI Exists in a Monomer-Dimer Equilibrium.

Our first objective was to express and purify the AceI transporter of *A. baumannii*. For this, we used an AceI construct containing amino acid residues 36 to 179, with a C-terminal hexahistidine tag, since this construct when expressed in *Escherichia coli* confers resistance to chlorhexidine (6). This truncated form of AceI (referred to as AceI for simplicity) was purified in *n*-dodecyl-β-d-maltopyranoside (DDM) and MS measurements in various detergents were performed. Well-resolved native mass spectra in *n*-dodecyl-*N*, *N*-dimethylamine-*N*-oxide (LDAO) indicates the presence of two different charge state series that correspond to monomers and dimers in solution (*SI Appendix*, Fig. S1*B*). The monomeric form is predominant under these conditions. The experimental masses for the monomer and dimer were 17,460 ± 1 Da and 34,922 ± 2 Da, respectively, which coincide with the theoretical monomer (17,461.80 Da) and dimer (34,923.6 Da) masses (*SI Appendix*, Table S1).

To explore the possibility that detergents might affect the monomer-dimer populations in solution, we acquired data for the detergents DDM, octyl glucose neopentyl glycol (OGNG), and *n*-octyl-β-d-glucopyranoside (OG). Both monomers and dimers can be observed in all detergents tested, with the highest population of dimers observed in OGNG (*SI Appendix*, Fig. S2) Our results suggest that this AceI construct exists in a monomer-dimer equilibrium in detergent micelles, in good agreement with the recent observations that purified AceI exhibits monomer and dimer populations on sodium dodecyl sulfate polyacrylamide gel electrophoresis (6, 9).

An important point to consider here is the potential influence of the first 35 amino acids on the oligomeric state of this protein. Since full-length AceI protein is difficult to express in *E. coli* (6), we made two BRIL (apocytochrome *b*_562_RIL)-fusion constructs, corresponding to the full-length protein (residues 1 to 179) and the truncated protein (residues 36 to 179). Following expression and isolation, we recorded the native mass spectra of these two constructs under similar conditions (*SI Appendix*, Fig. S3). In both cases, the mass spectra show that the two charge state distributions for monomeric and dimeric species are similar to the construct used above containing amino acid residues 37 to 179 with a hexahistidine-tag. This result suggests that the first 35 residues at the N terminus are not necessary for dimer formation.

We hypothesized that this monomer-dimer equilibrium might be lipid-mediated, as lipids have been shown to influence higher-order oligomer formation (27). We incubated the protein (5 µM AceI in 200 mM ammonium acetate, pH 6.8 with 0.05% LDAO) with phospholipids, including phosphatidylethanolamine (PE), phosphatidylglycerol (PG), and cardiolipin (CDL), at a final concentration of 20 µM and recorded mass spectra under optimized conditions (*SI Appendix*, Fig. S4). Lipids were observed to bind to both monomers and dimers. Compared with apo-AceI, the relative ratio of monomer to dimer was not significantly increased in the cases of PE and PG; however, in the case of CDL, more dimers were observed compared with monomers, suggesting that the presence of CDL with two phospholipid head groups may act to bridge the two subunits. Analogous to previous cases, such as LeuT and NhaA (27), we speculate that this bridging occurs by interacting with conserved arginine residues (R70) present in loops between transmembrane helices.

### pH Dependence on the Monomer-Dimer Equilibrium of AceI.

**Fig. 1.**
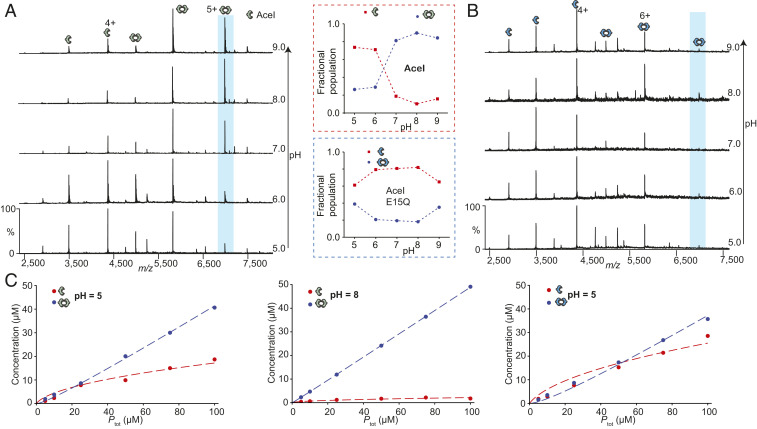
Effect of pH on monomer-dimer equilibrium. (*A*) Mass spectra of 10 µM wild-type AceI in different solutions from pH 5 to pH 9. The dimer population increases as the pH of solution increases (spectra colored in red). (*B*) Mass spectra of 10 µM E15Q AceI mutant at different pH values. No change in the monomer-dimer population is observed. (*Inset*) Monomer-dimer ratios at different pH values. (*C*) At different protein concentrations, a plot of observed monomer and dimer concentrations vs total protein concentration. Wild-type AceI at pH 5 (*Left*) and pH 8 (*Middle*); E15Q mutant at pH 5 (*Right*).

We next sought to investigate the effect of pH on monomer-dimer equilibrium. We acquired mass spectra of AceI at different solution pH values, ranging from 5 to 9, at a protein concentration of 10 µM. At low pH (5 and 6), we observed populations of monomers and dimers. As the pH was increased from 7 to 9, the resulting mass spectra revealed predominantly dimeric species (Fig. 1*A* and *SI Appendix*, Fig. S5 *A* and *B*). This observation indicates that the monomer-dimer equilibrium is changed during the deprotonation of charged residues.

To quantify the pH-induced changes, we performed titration experiments by changing the total protein concentration from 5 to 100 µM and changing pH values from 5 to 9 and recording the resulting mass spectra (*SI Appendix*, Fig. S5 *A* and *B*). The concentrations of monomer and dimer at each protein concentration were determined, and the monomer-dimer equilibrium binding constant (*K*_D_) at different pH values was plotted against the total protein concentration (*SI Appendix*, *SI Materials and Methods*) (33). The *K*_D_ value for the dimer at pH 5 was 7.13 µM, 70-fold higher than the value obtained at pH 8 (*K*_D_ = 0.10 µM) (Fig. 1*C* and *SI Appendix*, Table S2). A comparison of K_D_ values at pH 6, 7, and 9 suggests that AceI exists predominantly as a dimer above pH 7, consistent with the deprotonated state of the protein being dimeric in solution.

The change in dimerization as a function of pH implies that a critical aspartate or glutamate side chain is deprotonated. Previous studies have implicated E15Q AceI as a nonfunctional mutant and have suggested that this residue has an indirect role in drug efflux (6). To investigate its role in dimerization and drug binding, we expressed and purified the E15Q AceI mutant using similar protocols as used for the wild-type protein. We obtained well-resolved mass spectra using similar MS conditions. Analogous to wild-type AceI at pH 6.8, the E15Q is in equilibrium that favors the monomer (*SI Appendix*, Fig. S6).

Since we observed a significant effect of pH on the monomer-dimer equilibrium for the wild-type protein, we carried out analogous experiments with the E15Q mutant. We recorded mass spectra of 10 µM E15Q AceI protein at different pH values from 5 to 9. Interestingly, in contrast to the wild-type protein, the E15Q mutant showed no significant changes in monomer-dimer ratios (Fig. 1*B*). This implies that the changes we observed for wild-type AceI result from the deprotonation of Glu15. In addition, at all pH values tested, the E15Q spectra appeared similar to the wild-type AceI spectrum at low pH, further demonstrating that this mutant is a good mimic of the fully protonated transporter. This suggests that the protonation/deprotonation of Glu15 plays an important role in transporter function. The previous observation that this mutant protein is nonfunctional (6) along with this finding of impaired dimer formation of the E15Q mutant imply that the protein is functional as a dimer.

We performed analogous titration experiments on the E15Q mutant protein as for the wild-type protein and plotted monomer and dimer concentrations against total protein concentration (*SI Appendix*, Fig. S5*C*). We calculated E15Q *K*_D_ values of 17.50 µM at pH 5 and 18.41 µM at pH 6. These *K*_D_ values are higher than those for the wild-type protein (7.13 µM at pH 5 and 5.69 µM at pH 6), indicating that E15Q populates the monomeric state at both low pH and high pH (Fig. 1*C* and *SI Appendix*, Table S2).

### Specificity of Chlorhexidine Binding and Increase in AceI Dimer Formation.

**Fig. 2.**
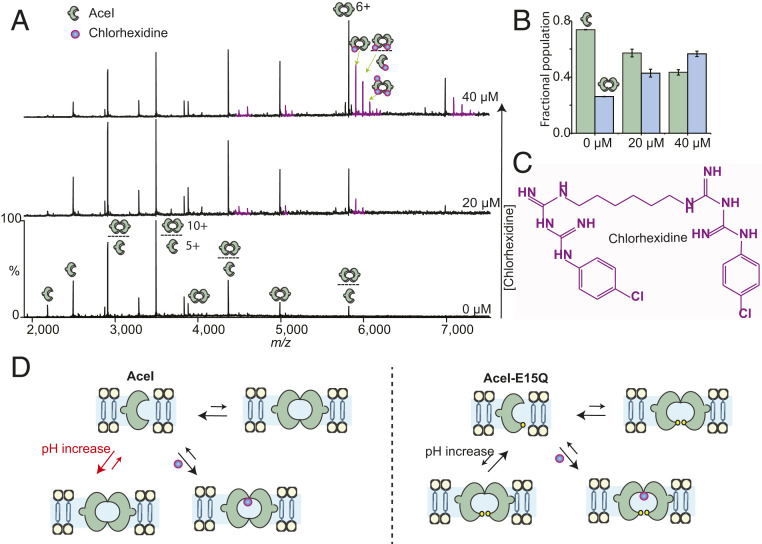
Chlorhexidine binding strengthens dimer formation. (*A*) Mass spectra of 5 µM AceI with increasing amounts of chlorhexidine. Additional peaks next to the main charge state distribution correspond to the mass of chlorhexidine. The number of chlorhexidine binding events >2 is considered nonspecific due to the use of high concentrations of chlorhexidine (top spectrum, 40 µM). Chlorhexidine binding increases the dimer population. (*B*) Relative ratios of the monomer-dimer population indicate that chlorhexidine strengthens the dimeric state. Error bars represent SDs (*n* = 3). (*C*) Structure of chlorhexidine. (*D*) Summary of AceI wild-type and E15Q data separated by a vertical line. Both AceI and E15Q exist in monomer-dimer equilibrium. The dimer population is significantly increased by a change in pH increase in wild-type, while E15Q remains in mostly a monomeric state, suggesting that deprotonation of this residue is critical for AceI function. The similar levels of chlorhexidine binding to both wild-type and E15Q mutant suggest that this residue is not involved in drug recognition.

Recent studies have investigated the ability of a large number of PACE protein homologs from different bacteria to bind to other biocides, antibiotics, and antimicrobial dyes (8). Most of the PACE proteins have been shown to confer resistance to one or more of these compounds, suggesting that PACE family proteins are multidrug efflux transporters. The binding properties of these compounds and structural changes involved during the transport are largely unknown. To further test whether *A. baumannii* AceI is also capable of recognizing multiple substrates, we recorded mass spectra of 5 µM AceI protein in the presence of 50 µM biocides, including benzalkonium, acriflavine, proflavine, and dequalinium. Interestingly, we did not observe any binding of these compounds to AceI. These results suggest that *A. baumannii* AceI is not able to bind to these biocides (*SI Appendix*, Fig. S7), consistent with the previous identification of *A. baumannii* AceI as a chlorhexidine efflux pump (6, 8). However, when tested with chlorhexidine, we observed multiple low-occupancy binding of up to three chlorhexidine molecules, indicative of a transport mechanism rather than a single specific binding interaction (Fig. 2 *A* and C).

Interestingly, in addition to chlorhexidine binding, our data also showed a significant increase in the dimer population when chlorhexidine was added to the protein. To investigate this further, we recorded mass spectra of AceI in the presence of increasing concentrations of chlorhexidine (Fig. 2*A*). A plot of relative ratios of monomer and dimer indicates that the addition of chlorhexidine increased dimer formation (Fig. 2*B*). We then carried out analogous experiments with the E15Q mutant and found that the binding of chlorhexidine also increased the dimer population of the mutant (*SI Appendix*, Fig. S6). This result indicates that chlorhexidine binding is important for the dimerization of the inactive E15Q mutant and further demonstrates that the E15Q residue is not involved in drug binding.

Taken together, the foregoing results suggest that the functional form of the protein is a dimer, and that the assembly of the dimer is mediated by binding of chlorhexidine and promoted by high pH conditions (Fig. 2*D*). The assembly of this pump is likely in response to an onslaught of high concentrations of chlorhexidine and is likely controlled by changes in cellular pH. The ability of bacteria to survive and grow at alkaline pH values has been recognized (34), which, together with the high concentrations of chlorhexidine, could promote assembly of the AceI dimer synergistically under alkaline conditions to export chlorhexidine.

### AceR Binds to Chlorhexidine and DNA.

Having studied the assembly of AceI protein in response to pH, lipids, and chlorhexidine assault, we set out to understand the regulation of AceI expression by AceR. We first attempted to express full-length AceR but found that the purified protein was unstable in solution. This observation is consistent with other LTTR proteins, in which relatively few studies are performed with full-length proteins owing to their poor solubility (13). To overcome this, we generated a construct with a Bril fusion at the N terminus of full-length AceR (AceR^FL^). This construct expressed the fusion protein in high yields, and the resultant protein was both soluble and stable. Mass spectra of this AceR^FL^ protein in 200 mM ammonium acetate at pH 8.0 showed the presence of two charge state distributions, one corresponding to dimeric AceR^FL^ and a lower-intensity series corresponding to tetrameric AceR^FL^. The measured mass of the dimer closely matches that of the calculated mass (*SI Appendix*, Table S1) and the dimer population is predominant (*SI Appendix*, Fig. S8*B*).

For comparison, we also produced a construct containing only the inducer-binding domain of AceR (amino acid residues 90 to 297 AceR^CTD^). We acquired mass spectra of this protein in 200 mM ammonium acetate (pH 8.0). The mass spectrum revealed a charge state distribution that corresponds to dimer formation, in good agreement with what has been observed for other LTTR family proteins (35).

As mentioned above, the function of LTTRs is defined by their ability to bind to the inducer molecule at the C terminus and to DNA at the N-terminal domain. To test whether AceR is able to bind to chlorhexidine, we incubated AceR^FL^ with 10 µM chlorhexidine and recorded mass spectra. We observed multiple adduct peaks adjacent to the main AceR^FL^ peaks in the mass spectra, corresponding to the binding of chlorhexidine (*SI Appendix*, Fig. S8*D*). To quantify the binding affinity, we recorded mass spectra of AceR^FL^ supplemented with various concentrations of chlorhexidine from 0 to 60 µM. The measured *K*_D_ value for chlorhexidine binding to AceR^FL^ was ∼24 ± 13 µM. For AceR^CTD^, the protein was more difficult to analyze by MS, owing to poor solubility in MS-compatible buffers; therefore, we used isothermal titration calorimetry. In this case, the *K*_D_ was ∼16 ± 5.5 µM (*SI Appendix*, Fig. S8*E* and Table S3). This suggests that the *K*_D_ values for chlorhexidine binding to AceR^FL^ and AceR^CTD^ are comparable and similar to values reported previously for AceR^CTD^ measured by a fluorescence quenching assay, as well as for other LTTRs measured using a variety of biophysical approaches (10, 20, 36, 37).

**Fig. 3.**
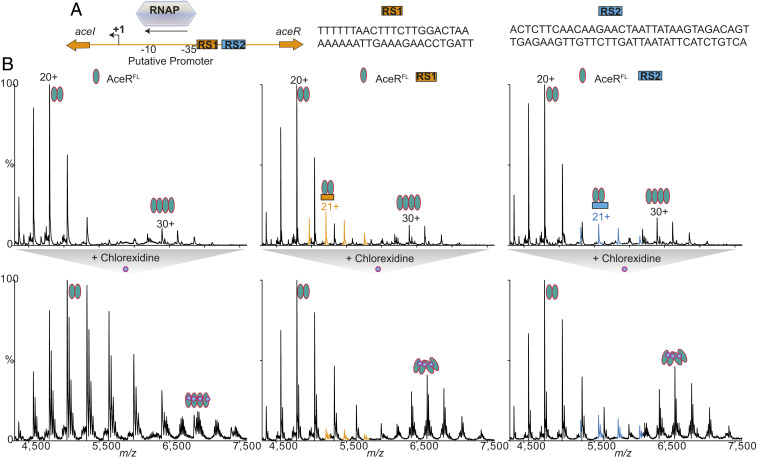
Chlorhexidine induces the tetramerization of AceR^FL^ in the presence of DNA. (*A*) schematic representation of the *aceI* promoter and regions of the DNA protected from DNase I digestion by AceR (10). +1, transcription start site. The −35 and −10 regions of the *aceI* putative promoter and sequences of recognition sites RS1 and RS2 are shown. (*B*, *Top*) The spectra of AceR^FL^ without DNA (*Left*) and with DNAs containing sites 1 (*Middle*) and 2 (*Right*). In the case of RS1 and RS2, a subpopulation of dimeric AceR^FL^ binding to DNA can be observed. (*B*, *Bottom*) When chlorhexidine was added to these solutions, in addition to chlorhexidine binding to dimers and tetramers, a significant increase in tetramerization of AceR can be seen in RS1 and RS2 (*Middle* and *Right*), while no such change is observed when no DNA is present (*Left*), suggesting that chlorhexidine binding in the presence of DNA induces the tetramerization of AceR.

Previous studies have identified two sites on DNA where AceR is able to bind, located upstream from the *aceI* gene (10) (Fig. 3*A*). To test the DNA-binding capacity of AceR, we constructed two sets of double-stranded (ds) DNA corresponding to the upstream *ace1* gene sequence containing the previously identified site 1 (22 nt) and site 2 (34 nt), designated RS1 (recognition site 1) and RS2. We incubated 2 µM RS1 with 2 µM AceR^FL^ in 200 mM ammonium acetate (pH 8.0) and recorded the mass spectra. The resulting spectrum shows an additional charge state distribution adjacent to the dimer distribution, with a mass corresponding to the binding of one RS1 to an AceR^FL^ dimer (Fig. 3*B*). Similar results were obtained with the DNA that included the RS2 site (Fig. 3*B*).

### Chlorhexidine Promotes Tetramerization of AceR in the Presence of DNA.

Activation of LTTRs is thought to result from conformational changes that occur when the inducer molecule binds to their inducer-binding domain and alters the DNA binding/bending. After characterizing the binding of chlorhexidine and DNA to AceR^FL^ separately, to further explore the role of chlorhexidine in the regulatory properties of AceR, we added 25 µM of chlorhexidine to the preincubated RS1-AceR^FL^ complex and recorded mass spectra (Fig. 3*B*). The spectrum showed several interesting features: (i) in addition to dimeric and tetrameric AceR^FL^, chlorhexidine binding was also observed for the dimeric AceR^FL^-RS1 complex; (ii) the amount of DNA binding to dimeric AceR^FL^ was reduced in the presence of chlorhexidine; and (iii) the population of chlorhexidine-bound AceR^FL^ tetramers was increased. These results suggest that chlorhexidine binding at the inducer-binding domain causes a quaternary structural change that favors interactions between AceR dimers to form tetramers. We did not directly observe the binding of DNA to tetramers, possibly due to the transient nature of this complex or to the changes induced in the overall structure of both AceR and DNA on chlorhexidine binding.

To further confirm the foregoing observations, we performed analogous experiments with RS2 (Fig. 3*B*). In this case, we noticed a comparable increase in the population of AceR^FL^ tetramers to that found earlier. In contrast to the analogous experiments with RS1, we observed relatively no change in the population of AceR^FL^-RS2 complexes with or without chlorhexidine. The absence of a significant change in the presence of chlorhexidine suggests that the AceR^FL^ interaction with RS2 is stable without the presence of the inducer molecule. Taken together, these results imply that RS2 is the RBS for AceR^FL^, in line with the known high-affinity binding sites for other LTTRs (13).

### Chlorhexidine Binding to AceR Facilitates RNA Polymerase Binding to Its Promoter.

To explore the regulation and activation events of the *aceI* gene, we studied the role of AceR^FL^ in the RNAP interactions with promoter DNA. We first characterized *E. coli* RNAP containing the σ^70^ factor using native MS and observed a charge state distribution consistent with the intact protein complex including all subunits (α_2_ββ′ωσ^70^) (*SI Appendix*, Fig. S9). The measured mass (460,893 ± 19 Da) closely coincides with the calculated mass (460,048 Da) (*SI Appendix*, Table S1), and the mass difference could correspond to two additional nucleoside diphosphates. In addition, we also observed subcomplexes with fewer subunits—namely ββ′ω, ββ′ωσ^70^, and α_2_ββ′σ^70^—in line with the loss of peripheral subunits from the large core formed from subunits ββ′.

**Fig. 4.**
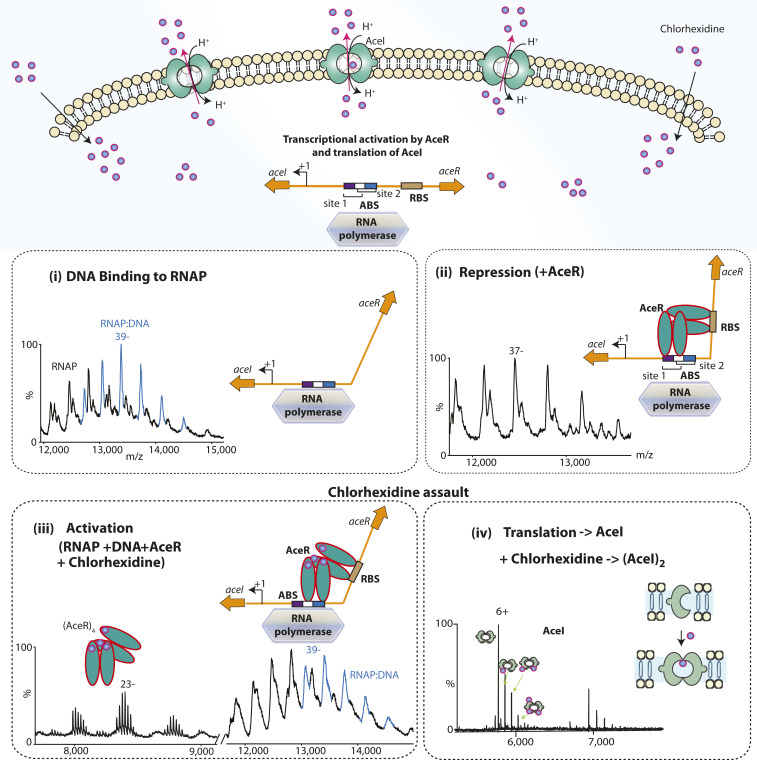
Roles of AceR and chlorhexidine in controlling the expression of *aceI* gene. (*Top*) Schematic illustration of transcriptional activation of the *aceI* gene by AceR in response to external chlorhexidine stimuli. The resulting AceI protein effectively dimerizes to efflux chlorhexidine. (*Bottom*) The individual steps during transcriptional regulation of the *aceI* gene by AceR. (i) Mass spectra of intact RNAP with promoter DNA showing that RNAP binds to the promoter DNA in 1:1 ratio in the absence of regulatory protein AceR; (ii) mass spectra of RNAP with the promoter DNA in the presence of AceR showing that the complex between RNAP and DNA is disrupted; (iii) mass spectra of RNAP with DNA and AceR and in the presence of chlorhexidine showing the presence of a 1:1 complex between RNAP and DNA and a tetrameric population of AceR; (iv) further translation results in the AceI protein, which dimerizes on binding to chlorhexidine and at high pH to efflux the excess cellular chlorhexidine.

To determine whether RNAP is able to bind to the promoter region in the absence of AceR^FL^, we first incubated RNAP with the 100-bp dsDNA comprising the promoter region of the *aceI* gene and including the RS1 and RS2 sites. Mass spectra showed a charge state series corresponding to a 1:1 complex between RNAP and the 100-bp DNA (Fig. 4 and *SI Appendix*, Fig. S9). Next, to understand the effect of the regulatory protein AceR on this DNA-RNAP complex, we incubated AceR^FL^ with the 100-bp DNA and RNAP complex formed as above and recorded mass spectra under similar conditions as before. The resulting mass spectrum showed charge state distributions that could be assigned to an individual AceR^FL^ dimer and the RNAP assembly without DNA. These observations suggest that the RNAP-DNA complex has released DNA (Fig. 4 and *SI Appendix*, Fig. S10). Thus, AceR^FL^ prevents transcription of the *aceI* gene by disrupting interactions between the promoter DNA and RNAP. Interestingly, we did not observe direct interactions between AceR^FL^ and RNAP, in contrast to other LTTRs, where the α-CTD of RNAP is known to interact with LTTR proteins (18, 19). That this RNAP-DNA complex is no longer viable in the presence of AceR^FL^ is indicative of the repressive nature of the protein on *aceI* transcription.

Since AceR^FL^ prevents *aceI* transcription by disrupting the DNA interactions with RNAP, we were intrigued to discover the effects of adding the inducer chlorhexidine to AceR^FL^ in the presence of RNAP and the 100-bp DNA. Mass spectra revealed several interesting features: (i) the charge state distributions corresponding to RNAP alone and the 1:1 complex of RNAP with DNA were readily discerned, and (ii) a significant increase in the tetramerization of AceR^FL^ was observed (Fig. 4 and *SI Appendix*, Fig. S10). This suggests that conformational changes in AceR^FL^ induced by chlorhexidine binding effect the release of DNA from interactions with AceR^FL^ to facilitate the recruitment of RNAP to the promoter for initiation of transcription. Consequently, under conditions of chlorhexidine assault, binding of the small molecule to regulatory protein releases the gene for transcription to initiate the protein production of AceI for its subsequent assembly in the inner membrane as a dimeric pump for export of chlorhexidine.

## Conclusions

The results of this study demonstrate that *A. baumannii* AceI exists in a monomer-dimer equilibrium in solution that is modulated by pH, cardiolipin, and chlorhexidine binding. Interestingly, binding of other cationic biocide compounds, including benzalkonium, acriflavine, and proflavine, was not observed, reaffirming that AceI binds specifically and confers resistance primarily to chlorhexidine (6). Analogous pH-induced dimer formation was not observed for the inactive AceI mutant E15Q, although chlorhexidine binding was not compromised significantly by this mutation.

A similar monomer-dimer equilibrium was also observed for EmrE, a well-studied SMR family member, on MS analysis (38). Structural information obtained from cryo-electron microscopy, X-ray crystallography, and nuclear magnetic resonance (NMR) spectroscopy, along with other experimental data, have established that EmrE functions as an asymmetric antiparallel dimer (39–48). For EmrE, an essential Glu14 residue (sharing conservation with Glu15 in AceI), is involved in drug-proton binding during efflux. Our results show that the E15Q AceI mutant displays a dramatically different pH response compared the wild-type AceI protein. While it is tempting to suggest, by analogy with EmrE, that AceI also forms a functional asymmetric dimer with E15 involved in proton recognition, high-resolution structural NMR spectroscopy and X-ray data are needed to support this idea. Moreover, the identification of key residues involved in drug and proton binding, together with their respective *pK*_a_ values, is needed to shed light on transport mechanisms. Nonetheless, collectively our present results suggest that similar to SMR family protein protonation, deprotonation of AceI transporter plays important roles in dimerization and efflux mechanisms and may motivate many follow-on experiments.

Turning to the regulation of *aceI* transcription via RNAP interactions with the gene, our data, along with previously reported results (10), have allowed us to propose a model for AceR-mediated transcription of the *aceI* operon. The following sequence of events is proposed: (i) RNAP binds to the promoter DNA in the absence of the regulatory protein AceR; (ii) this interaction is disrupted when AceR binds DNA, and in the absence of chlorhexidine, AceR binds to at least two sites of DNA as AceR dimers; (iii) when AceR interacts with chlorhexidine, it undergoes a conformational change; the tetrameric form either releases the DNA or shifts the position of the DNA-binding region to allow RNAP to bind onto the promoter DNA to proceed with *aceI* transcription.

In summary, the results reported here reveal mechanistic details governing the assembly of this PACE family transporter modulated by acid-base chemistry of a conserved anionic residue and by ligand binding. It is interesting to consider these results in the light of the pH range for chlorhexidine efficacy (pH 5 to 7) and the observation that the pH of chronic wounds increases to alkaline values (49), and accordingly, it is tempting to speculate that infection of chronic wounds with *A. baumannii* would lead to conditions in which formation of the AceI efflux pump would be favored, due not only to the high pH conditions but also to the availability of CDL in the inner membrane of this gram-negative bacteria (50). Adding to these conditions the high concentration of chlorhexidine, which further promotes dimerization of the AceI pump, as well as the increased transcription of the *aceI* gene following chlorhexidine binding to the regulatory protein AceR, and it is easy to see how these conditions conspire to efflux chlorhexidine with maximum efficiency. Therefore, inhibiting dimerization of the pump by controlling pH conditions, as well as preventing drug efflux through the use of inhibitors or blocking chlorhexidine-binding sites on AceR, would lead to the prevention of chlorhexidine resistance. More generally, knowledge of these mechanisms contributes to our understanding of the modes of action of the secondary active transporter family and thereby aids efforts to combat the worldwide problem of bacterial antibiotic resistance.

## Methods

Before MS analysis, soluble proteins were buffer-exchanged into 200 mM ammonium acetate pH 8.0, and membrane proteins were buffer-exchanged into 200 mM ammonium acetate at various pH values, with twice the CMC (critical micelle concentration) of the detergent of interest. Data were collected on a modified QExactive hybrid quadrupole-Orbitrap mass spectrometer (Thermo Fisher Scientific) optimized for analysis of high-mass complexes, using methods previously described for membrane proteins (25). Data were analyzed using Xcalibur 3.0 (Thermo Fisher Scientific) and UniDec software (51). The relative intensities of monomers and dimers were obtained by deconvoluting the native MS data using UniDec and were converted to mole fractions to determine the monomer and dimer concentrations at equilibrium. To obtain the monomer-dimer equilibrium constants, a previously established monomer-dimer model was used (33). Detailed descriptions of the methodology for protein expression constructs, protein expression and purification, native mass spectrometry, and isothermal titration calorimetry are described in *SI Appendix*, *Materials and Methods*.

### Data Availability.

The raw data that support the findings of this study have been deposited in the Figshare database at https://figshare.com/articles/_/12253085.

## Supplementary Material

Supplementary File
