# Supplementary material for: Assembly and regulation of the chlorhexidine-specific efflux pump AceI
Source: Proc Natl Acad Sci U S A. 2020 Jul 7;117(29):17011–8. doi: 10.1073/pnas.2003271117 (PMC7382281; doi:10.1073/pnas.2003271117)
Supplement: Supplementary File [file pnas.2003271117.sapp.pdf]

Supporting Information (SI) Appendix for

Assembly and regulation of the chlorhexidine specific efflux pump AceI

Jani Reddy Bolla1, Anna C. Howes1, Francesco Fiorentino1, and Carol V. Robinson1, *

1. Physical and Theoretical Chemistry Laboratory, Department of Chemistry, University of Oxford, South Parks Road, OX1 3QZ, UK

* Corresponding author: Carol V. Robinson

Email: carol.robinson@chem.ox.ac.uk

This file includes:

Supplementary materials and methods

SI references

Figures S1 to S10

Tables S1 to S3

www.pnas.org/cgi/doi/10.1073/pnas.2003271117

SI Materials and Methods

Protein expression constructs:

The plasmids used for over-expression of the proteins in this study were obtained by inserting the respective gene fragment into a modified pET15b vector using an InFusion kit (Clonetech). The DNA sequences were verified by sequencing (Source Bioscience). All the proteins contain a 6×His tag at the C-terminus was overexpressed in *E. coli* C43(DE3) (Lucigen) cells, grown in LB medium containing ampicillin (100 μgml^{-1}). When the culture reached an absorbance at 600nm of ~0.5, expression was induced with 0.5mM isopropyl β-thiogalactopyranoside (IPTG) for 3h at 37 °C.

E15Q mutant of AceI was prepared by a PCR-based method, using the plasmid that was used for expressing wild type protein. Expression and purification of this mutant is done in the same manner as for the wild type.

Protein expression and purification:

Proteins were purified in a similar manner according to the following procedure at 4 °C. Cells were pelleted by centrifugation at 5,000g and resuspended in a buffer containing 50 mM Tris-HCl (pH 8.0), 300 mM NaCl and EDTA-free protease inhibitor cocktail (Roche). The cells were then disrupted by a microfluidizer (Microfluidics). After centrifugation (20,000g for 20 min), the supernatant was filtered and loaded onto a 5 ml His Trap-HP column in case of soluble proteins while for membrane proteins, the supernatant was ultracentrifuged (200,000g), and the membrane fractions were collected. The proteins were solubilized from the membrane fraction with 20 mM Tris (pH 8.0), 150 mM NaCl, 20% glycerol, 2% DDM (Anatrace) for 2 h at 4 °C. The insoluble material was removed by ultracentrifugation. The supernatant was filtered before loading onto a 5 ml His Trap-HP column (GE Healthcare) equilibrated in 20 mM Tris (pH 8.0), 150 mM NaCl, 20 mM imidazole, 10% glycerol and 0.03% DDM. After the clarified supernatant was loaded, the column was initially washed with 50 ml of 20 mM Tris (pH 8.0), 150 mM NaCl, 20 mM imidazole, 10% glycerol and 0.03% DDM and washed again with 50 ml of 20 mM Tris (pH 8.0), 150 mM NaCl, 80 mM imidazole, 10% glycerol and 0.03% DDM. The bound protein was then eluted with 20 mM Tris (pH 8.0), 150 mM NaCl, 500 mM imidazole, 10% glycerol and 0.03% DDM. Peak fractions were pooled, dialyzed and concentrated in a buffer containing 20 mM Tris (pH 8.0) and 150 mM NaCl, 10% glycerol and 0.03% DDM. Similar buffers were used for soluble protein purification without the DDM detergent. Concentrated protein was either used immediately or flash-frozen in liquid nitrogen and stored at −80 °C.

Native mass spectrometry:

Prior to MS analysis, soluble proteins were buffer exchanged into 200 mM ammonium acetate pH 8.0, while membranes were buffer-exchanged into 200 mM ammonium acetate at various pHs, with 2 × CMC (critical micelle concentration) of the detergent of interest using a Biospin-6 (BioRad) column and introduced directly into the mass spectrometer using gold-coated capillary needles (prepared in-house). Data were collected on a modified QExactive hybrid quadrupole-Orbitrap mass spectrometer (Thermo Fisher Scientific) optimized for analysis of high-mass complexes, using methods previously described for membrane proteins (1). The instrument parameters were as follows: capillary voltage 1.2 kV, S-lens RF 100%, quadrupole selection from 1,000 to 15,000 m/z range, collisional activation in the HCD cell 100–200 V, argon UHV pressure 1.12×10^{-9} mbar, temperature 60 °C, resolution of the instrument 17,500 at m/z = 200 (a transient time of 64 ms) and ion transfer optics (injection flatapole, inter-flatapole lens, bent flatapole, transfer multipole: 8, 7, 6 and 4 V, respectively). The noise level was set at 3 rather than the default value of 4.64. No in-source dissociation was applied. Baseline subtraction was performed for the data shown in Fig. 3 and *SI Appendix*, Fig. S2 using UniDec software (2), while all other data were analysed using Xcalibur 3.0 (Thermo Scientific). The relative intensities of monomers and dimers were obtained by deconvoluting the native MS data using UniDec and were converted to mole fraction to determine the monomer and dimer concentrations at equilibrium. To obtain the monomer-dimer equilibrium constants, a previously established monomer-dimer model was used (3). Similar parameters were used for data processing in UniDec when comparisons are made.

Lipids and antibiotics were diluted into a buffer containing 200 mM ammonium acetate and 0.05% (w/v) LDAO and were added in different ratios to solutions of AceI in the same buffer. All experiments were repeated three times.

Native *E. coli* RNAP (Creative Enzymes) was buffer exchanged into 200 mM ammonium acetate pH 8.0 before adding it to the $AceR^{FL}$ protein and 100 bp dsDNA. Data were collected on Q-Exactive UHMR Hybrid Quadrupole-Orbitrap mass spectrometer (Thermo Fisher) in –ve polarity with the exception that the capillary voltage was set 1.0 kV and with the similar parameters as above operating in –ve polarity.

Isothermal Titration Calorimetry

$AceR^{CTD}$ was prepared by dialyzing protein against 20 mM HEPES pH 7.0 buffer and the concentration was adjusted to 100 µM. Chlorhexidine was dissolved in the same buffer to a final concentration of 1mM, to avoid any heat of dilution mismatch. ITC reactions were carried out on a MicroCal PEAQ-ITC

Automated instrument (Malvern Panalytical) with a cell temperature of 25 °C, a stirring speed of 750 rpm and high gain feedback mode. 2 µl of ligand was injected each time into protein in the cell, with injections occurring at 180 s intervals and lasting 5 s each. Data were processed and analyzed using the integrated MicroCal ITC Data Analysis Software tool with heat production fitted to a one-site binding model. The binding experiments were performed in triplicate.

SI references

1. J. Gault et al., High-resolution mass spectrometry of small molecules bound to membrane proteins. Nat Methods **13**, 333-336 (2016).

2. M. T. Marty et al., Bayesian deconvolution of mass and ion mobility spectra: from binary interactions to polydisperse ensembles. Anal Chem **87**, 4370-4376 (2015).

3. L. A. Bergdoll et al., Protonation state of glutamate 73 regulates the formation of a specific dimeric association of mVDAC1. Proc Natl Acad Sci USA **115**, E172-E179 (2018).

SI Figures and Tables

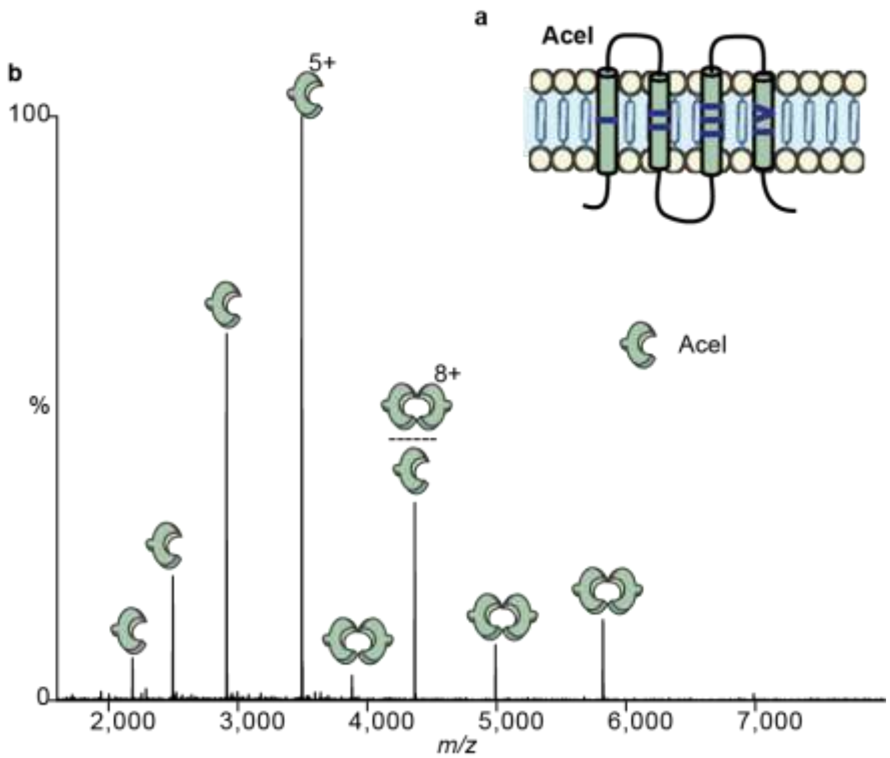

Fig. S1. Mass spectrum of AceI protein. a) Membrane topology of AceI protein, AceI consists of four transmembrane helices b) mass spectrum of AceI protein in LDAO, showing charge state series correspond to monomers and dimers. Observed and calculated masses for AceI protein monomers and dimers are listed Table S1

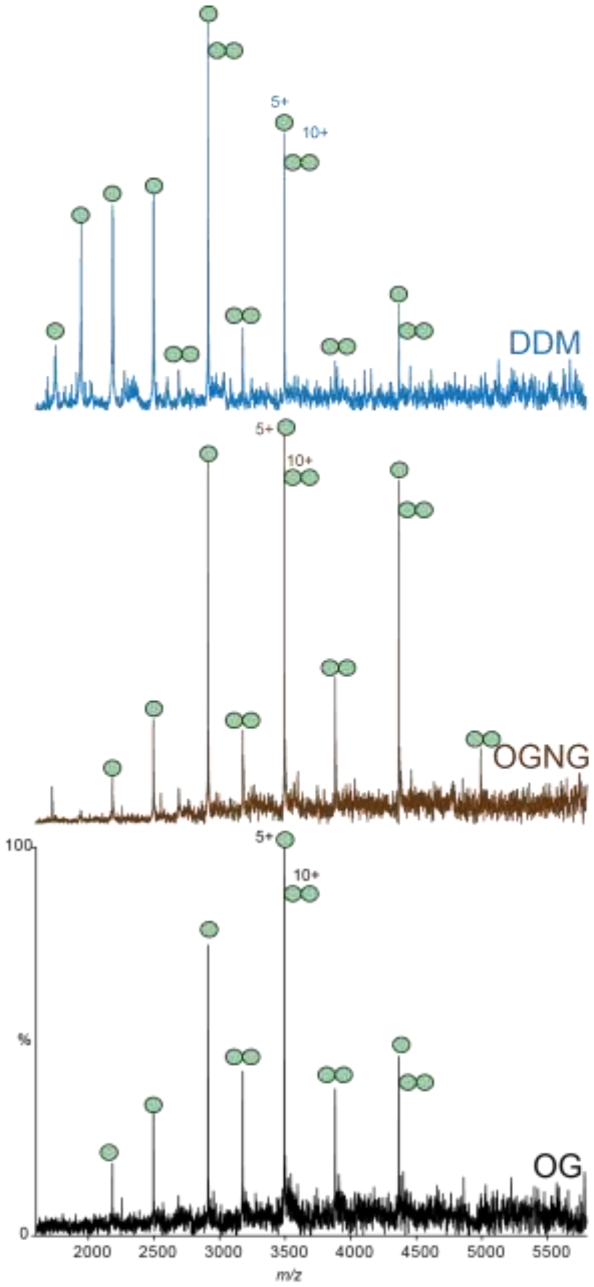

Fig. S2. Mass spectra of AceI in different detergents: Mass spectra of AceI in DDM, OGNG, and OG look similar to the spectrum in LDAO, in all detergents AceI seem to exist in a monomer-dimer equilibrium.

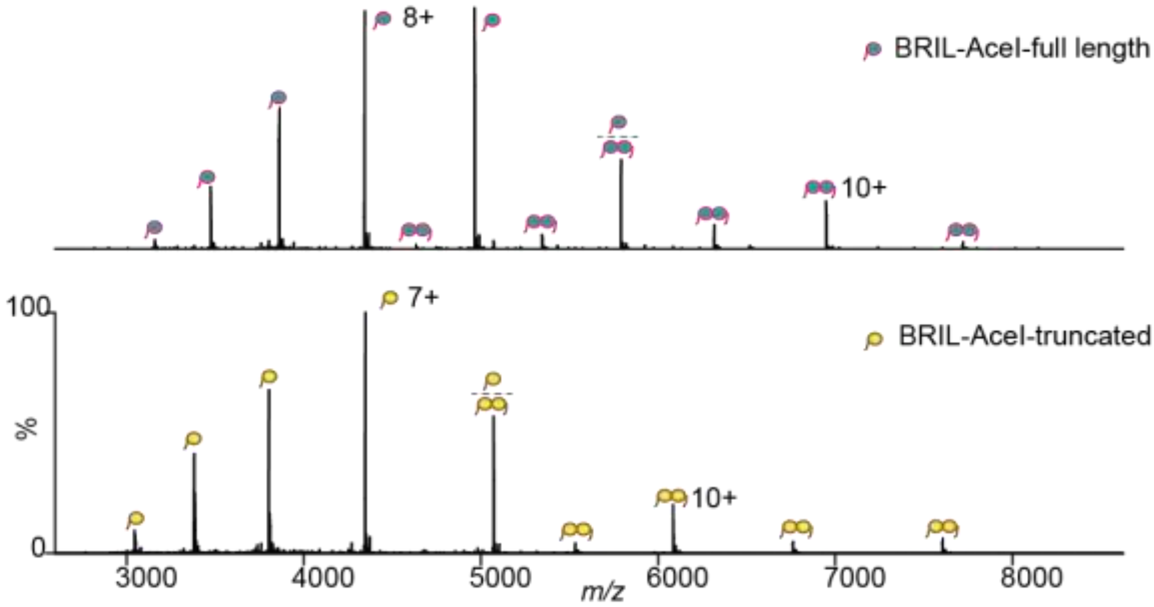

Fig. S3. Mass spectra of BRIL-fusion AceI constructs: Mass spectra of full length and truncated AceI with BRIL fusion partner also shows the existence of monomers and dimers in solution. This suggests that the N-terminal 35 residues are not important for dimer formation.

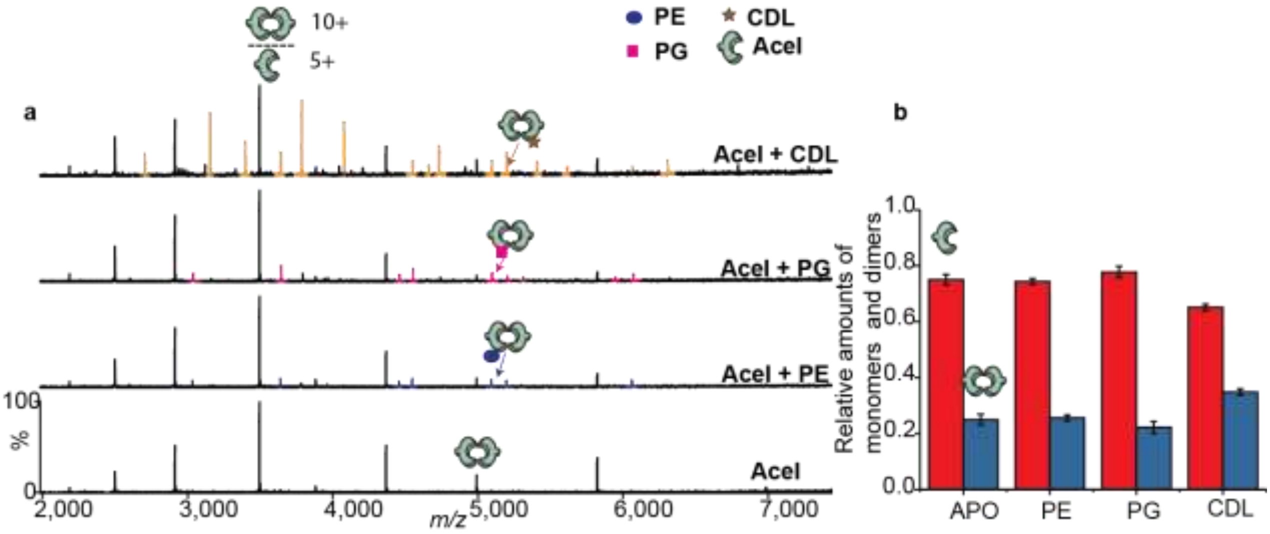

Fig. S4. Effect of lipids on monomer-dimer equilibrium: a) mass spectra of AceI supplemented with PE, PG, and CDL, lipid-bound peaks are observed in all cases, b) relative ratios monomer-dimer population show that increase in dimers population is observed only in case of CDL.

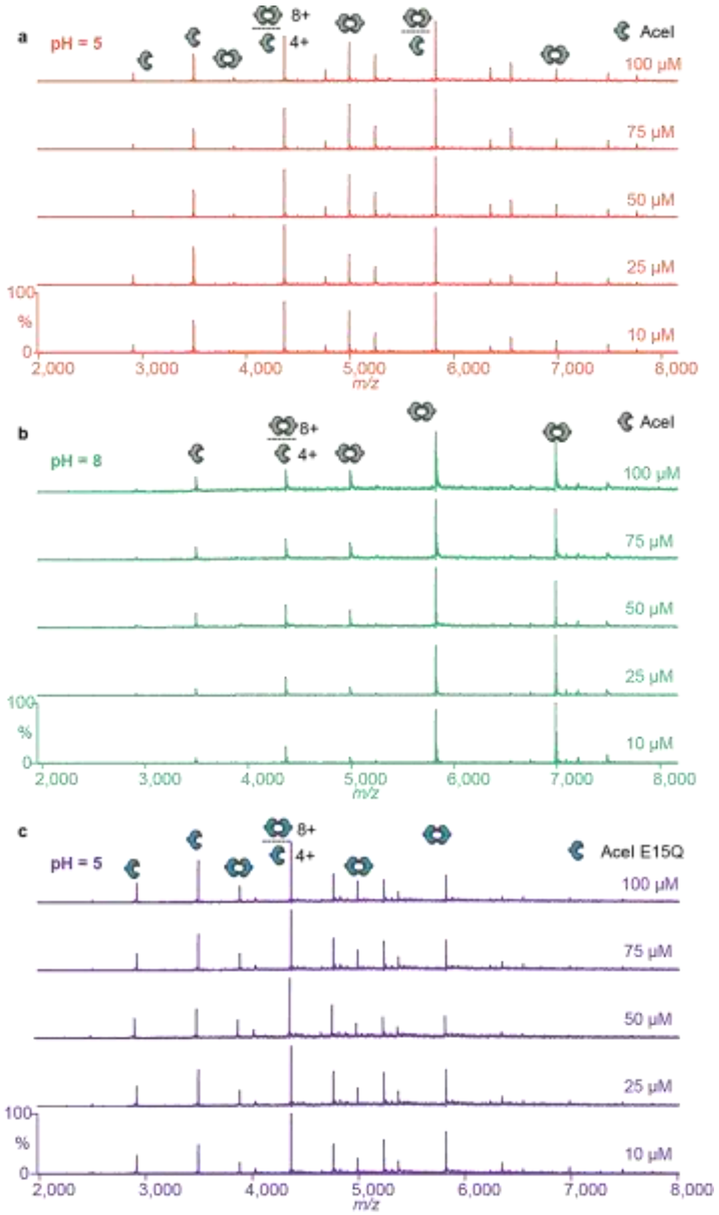

Fig. S5. Effect of pH on AceI wild type and E15Q mutant; raw data showing monomer-dimer populations at different pHs and at various protein concentrations ranging from 10 µM to 100 µM. Spectra for wild type protein at pH 5 and 8 are labelled with orange and green colour respectively while for E15Q the spectra are coloured in purple.

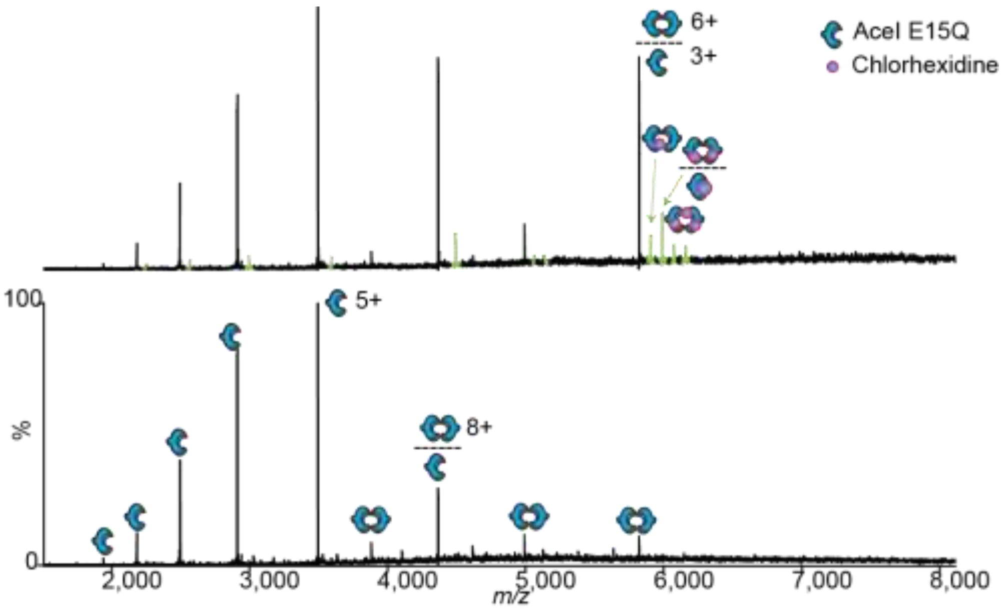

Fig. S6. Chlorhexidine binding to E15Q AceI mutant: mass spectra of E15Q mutant both in presence (top spectrum) and absence (bottom spectrum) of chlorhexidine. Presence of chlorhexidine peaks suggest that this mutant still be able to bind to chlorhexidine.

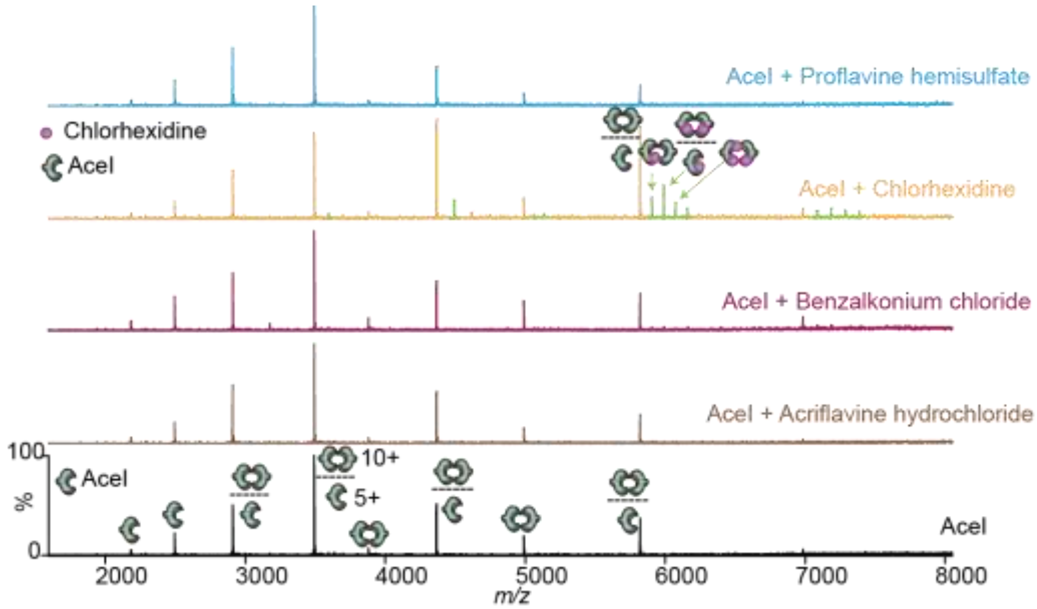

Fig. S7. Antibiotics binding to AceI: mass spectra of AceI in presence of various biocides supplemented at a concentration of 50 µM. Adduct peaks are observed only in case of chlorhexidine, suggesting that this protein specifically recognises chlorhexidine.

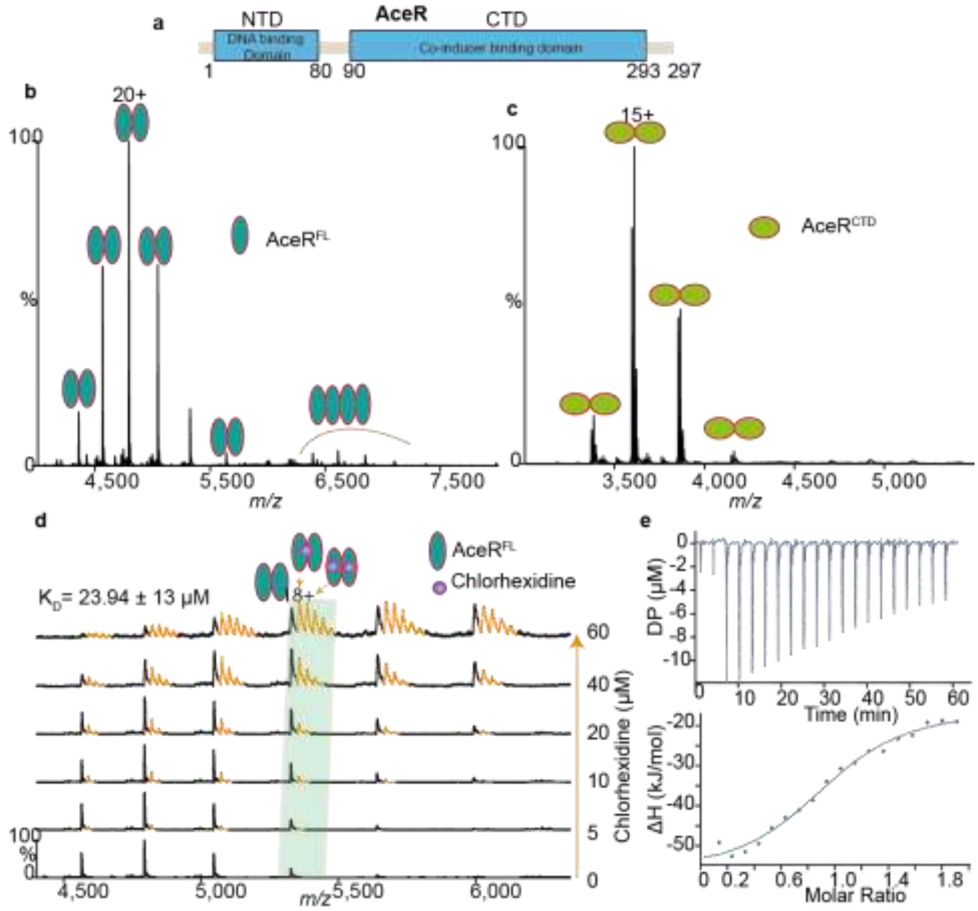

Fig. S8. Mass spectra of AceR^{FL} and AceR^{CTD} and binding of chlorhexidine; a) domain architecture of AceR, AceR contains N-terminal DNA binding domain and C-terminal inducer binding domain b) mass spectra of AceR^{FL} show that this protein exists in a dimer and tetramer equilibrium, c) mass spectra of AceR^{CTD} show that this protein is dimer in solution, d) titration of chlorhexidine binding to AceR^{FL}, chlorhexidine adduct peaks are highlighted in orange and range from 1 to 5 molecules at higher concentration of chlorhexidine tested, e) binding affinity of chlorhexidine to AceR^{CTD} was measured using ITC.

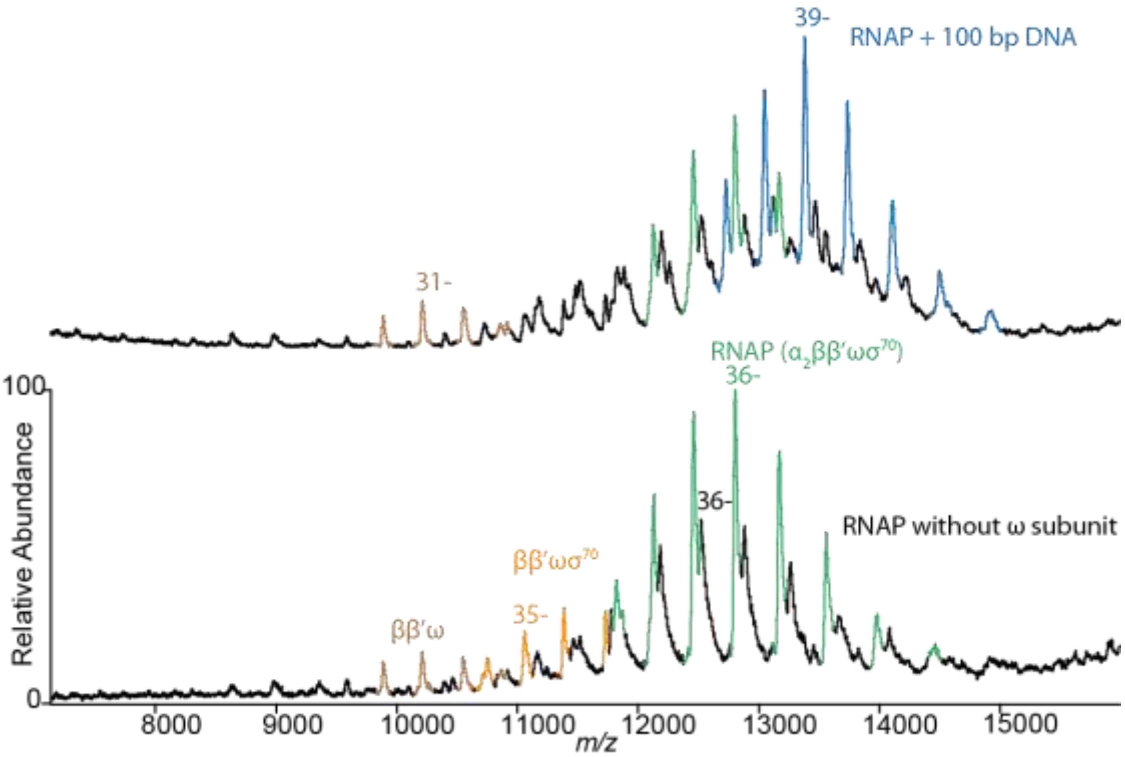

Fig. S9. RNAP binds to the *aceI* promoter DNA: mass spectra of RNAP, showing the presence of full complex with σ70 and other sub complexes lacking one or two subunits, bottom spectrum. The addition of 100 bp DNA that contains promoter region yields a new charge state distribution that corresponds to 1:1 complex of RNAP: DNA, top spectrum.

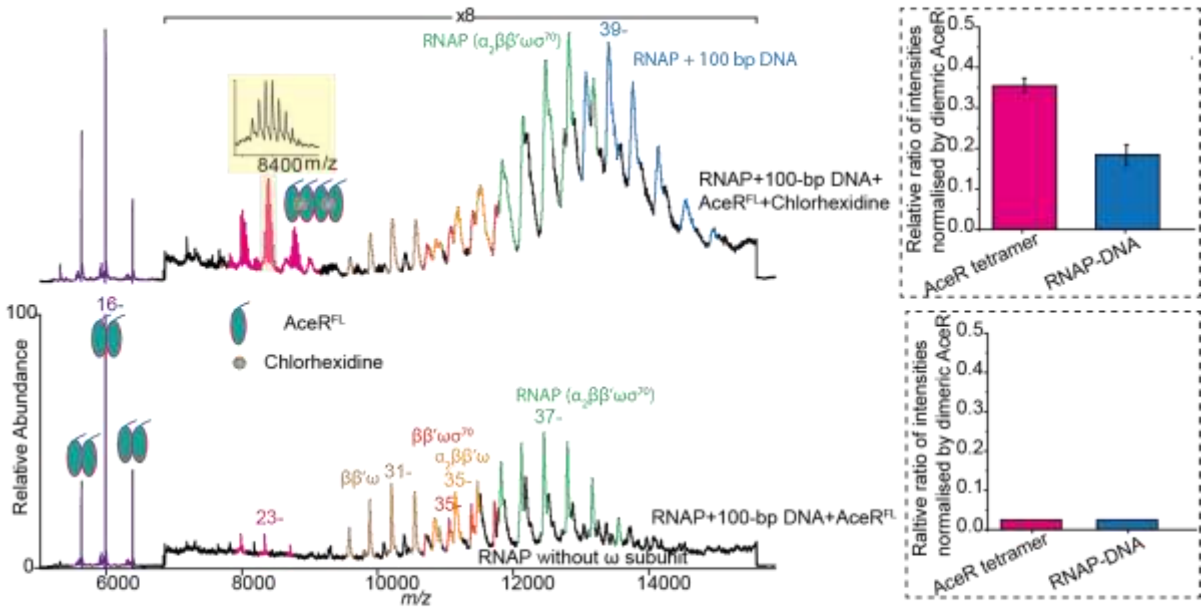

Fig. S10. Chlorhexidine-induced conformational changes facilitate the RNAP binding to promoter DNA. No interaction between RNAP and DNA in presence of AceR^{FL}, bottom spectrum, indicative of repressive nature of AceR. The addition of chlorhexidine significantly increases the tetramerisation of AceR^{FL}, which in turn promotes the interaction between RNAP and the promoter DNA, top spectrum. Relative ratios of AceR tetramer and RNAP-DNA complexes are shown in inserts, these clearly indicate that the increase in AceR tetramer formation is directly correlated with RNAP binding to promoter DNA.

Table S1. Measured and calculated masses. M – Monomer, D- dimer, and Te- tetramer

Protein	Calculated mass (Da)	Measured mass ± s.d. (Da)
AceI	17461.8 (M)	17460 ± 1 (M), 34922 ± 2 (D)
AceI-E15Q	17460.8 (M)	17459 ± 1.5 (M), 34921 ± 1 (D)
$AceR^{CTD}$	27102.9 (M)	54121 ± 1 (D)
$AceR^{FL}$	48044.8 (M)	95881 ± 1.4 (D), 191808 ±13 (Te)
RS1	13463.8	13484 ± 1
RS2	20877.6	20877.6 ± 0.3
$AceR^{FL}$+RS1	109553.4 (D+M)	109327 ± 0.3
$AceR^{FL}$+RS2	116965.6 (D+M)	116790 ± 15
100 bp DNA	59785.7	61067 ± 31
RNAP	460048.7	460893 ± 19
RNAP + 100 bp DNA	519834.4	521908 ± 15

Table S2. Monomer-dimer dissociation constants at different pH

**Apo**		
**pH**	**K_D µM**	**χ2**
5	7.13	16.15×10^{-12}
6	5.69	59.40×10^{-12}
7	0.92	9.40×10^{-12}
8	0.10	0.38×10^{-12}
**E15Q**		
pH		
5	17.50	44.33×10^{-12}
6	18.41	39.70×10^{-12}

Table S3. Binding parameters calculated for each ITC experiment of chlorhexidine binding to $AceR^{CTD}$

**Experiment**	1	2	3
**N (sites)**	1.04 ± 0.06	0.89 ± 0.06	0.96 ± 0.03
**K_D (μM)**	15.6 ± 5.4	22.8 ± 9.0	9.5 ± 2.3
**ΔH (kJ/mol)**	-37.7 ± 4.4	-41.2 ± 6.3	-39 ± 2.6
**ΔG (kJ/mol)**	-27.5	-26.5	-28.7
**-TΔS (kJ/mol)**	10.2	14.7	10.3
**Red. Chi-sqr. $(kJ/mol)^2$**	1.07	0.993	1.02
